# Effectiveness and cost-effectiveness of an electronic mindfulness-based intervention (eMBI) on maternal mental health during pregnancy: the *mindmom* study protocol for a randomized controlled clinical trial

**DOI:** 10.1186/s13063-020-04873-3

**Published:** 2020-11-17

**Authors:** Mitho Müller, Lina Maria Matthies, Maren Goetz, Harald Abele, Sara Yvonne Brucker, Armin Bauer, Johanna Graf, Stephan Zipfel, Lena Hasemann, Markus Wallwiener, Stephanie Wallwiener

**Affiliations:** 1 Ludwig-Maximilians-Universität München, Munich, Germany; 2 Universitätsklinikum Heidelberg Frauenklinik, Heidelberg, Germany; 3 Universitätsklinikum Heidelberg Zentrum für Kinder und Jugendmedizin, Heidelberg, Germany; 4 Universitäts-Frauenklinik Tübingen, Tübingen, Germany; 5 Institute for Women’s Health Tübingen, Tübingen, Germany; 6 Universitätsklinikum Tübingen Medizinische Universitätsklinik, Tübingen, Germany; 7 Universität Bielefeld, Bielefeld, Germany

## Abstract

**Background:**

Mental disorders are common during the peripartum period and may have far-reaching consequences for both mother and child. Unfortunately, most antenatal care systems do not provide any structured screening for maternal mental health. As a consequence, mental illnesses are often overlooked and not treated adequately. If correctly diagnosed, cognitive behavioral therapy is currently the treatment of choice for mental illnesses. In addition, mindfulness-based interventions (MBIs) seem to represent a promising treatment option for anxiety and depression during the peripartum period. Considering the internet’s increasing omnipresence, MBIs can also be offered electronically via a (tablet) computer or smartphone (electronically based MBI = eMBI).

**Objective:**

The current study aims to examine the clinical effectiveness and cost-effectiveness of an eMBI (the *mindmom* application) developed by an interdisciplinary team of gynecologists, psychologists, and midwives, teaching pregnant women how to deal with stress, pregnancy-related anxiety, and depressive symptoms. The study sample consists of pregnant women in their third trimester who screened positive for emotional distress. The *mindmom* study is a bicentric prospective randomized controlled trial (RCT), which is currently conducted at the University women’s hospitals of Heidelberg and Tübingen, Germany.

**Methods:**

Within the scope of the routine prenatal care, pregnant women attending routine pregnancy care in Baden-Wuerttemberg, Germany, are invited to participate in a screening for mental distress based on the Edinburgh Postnatal Depression Scale (EPDS). Women with an EPDS screening result > 9 will be referred to one of the *mindmom* coordinating study centers and are offered counseling either face-to-face or via videotelephony. After an initial psychological counseling, women are invited to participate in an eMBI in their last pregnancy trimester. The study will enroll *N* = 280 study participants (*N* = 140 per group), who are randomized 1:1 into the intervention (IG) or control group (treatment as usual = TAU).

All participants are requested to complete a total of 7 digital assessments (5 visits pre- and 2 follow-up visits postpartum), involving self-report questionnaires, sociodemographic and medical data, physiological measures, and morning cortisol profiles. The primary outcome will be depressive and anxiety symptoms, measured by the Edinburgh Postnatal Depression Scale, the State Trait Anxiety Questionnaire, and the Pregnancy-Related Anxiety Questionnaire. Secondary outcomes include mindfulness, satisfaction with birth, quality of life, fetal attachment, bonding, mode of delivery, and cost-effectiveness.

**Discussion:**

This is the first German RCT to examine the (cost-)effectiveness of an eMBI on maternal mental health during pregnancy. If successful, the *mindmom* app represents a low-threshold and cost-effective help for psychologically distressed women during pregnancy, thereby reducing the negative impact on perinatal health outcome.

**Trial registration:**

Deutsches Register Klinischer Studien, German Clinical Trials Register DRKS00017210. Registered on 13 January 2020. Retrospectively registered.

**Supplementary information:**

The online version contains supplementary material available at 10.1186/s13063-020-04873-3.

## Background

Mental disorders, especially anxiety and depressive disorders, are common during the peripartum period and may have far-reaching consequences for both mother and child [1–3]. The prevalence of peripartum depressive disorders is reported to be approximately 9% in Germany [4]. With prevalence rates ranging between 39% ante- and 16.5% postnatally, most studies indicate that women are even more susceptible to anxiety disorders during pregnancy [2, 3]. Furthermore, anxiety and depression have a very high comorbidity of approximately 50% [5].

Maternal psychological distress has been linked to adverse peripartum health outcomes for both mother and child, such as preterm delivery, low birth weight, maternal substance abuse in pregnancy, impaired mother-child interaction, childhood regulatory dysfunction, and impaired cognitive and psychomotor development [3, 6, 7].

Furthermore, the rising rate of cesarean delivery, which is currently estimated at 31% in Germany, is known to be associated with maternal mental disorders [4]. About 80% of indications for cesarean section are relative indications, of which 10% are due to increased levels of maternal anxiety. Effective treatment of peripartum psychological distress could thus have the potential to lower the cesarean section rate [8].

As most antenatal care systems worldwide do not provide any structured screening for maternal mental health, mental illnesses are often overlooked and not treated adequately. Recent studies have shown that peripartum depression is not detected in 80% of women, and only 20% of those affected receive an appropriate treatment [9]. This appears even more relevant, as maternal mood disorders can lead to substantial health care and societal costs. Furthermore, the treatment of peripartum mental disorders is particularly challenging, as medical treatment is often rejected for fear of harming the fetus [10, 11]. Thus, there is a high demand for integrating a validated screening for maternal mental distress in routine antenatal care and for easily accessible non-medical treatment options during pregnancy.

### Physiology of stress and neurobiological correlates

The high vulnerability for experiencing mental distress during the peripartum period can partly be attributed to the hormonal changes accompanying pregnancy and birth. Studies have repeatedly discussed associations with prolactin, gonadotropins, steroids, thyroid-stimulating hormone, and cortisol [2, 3]. One promising explanation is that mental disorders such as depression and anxiety are linked to changes in hypothalamic-pituitary-adrenocortical axis (HPAA) activity. In a current review, Szpunar and Parry identified significant correlations between morning cortisol levels and the presence of major depression [2, 3]. Furthermore, women with depressive and/or comorbid anxiety disorders during pregnancy respond to stress with a significantly higher cortisol release than healthy controls [12, 13].

During pregnancy, fetal cortisol concentrations are linearly associated with maternal blood concentrations [14, 15]. Thus, authors of the so-called fetal programming hypothesis postulated that elevated cortisol levels during pregnancy could possibly have continuing aftermath on the fetal environment through structural changes in the limbic system (e.g., hippocampus or amygdala) as well as in limbic regions of the cortex [16, 17]. Further studies on the predictive significance of daily maternal cortisol profile curves for the course of pregnancy and childhood development are urgently needed.

### Mindfulness-based interventions

Currently, cognitive behavioral therapy represents the treatment of choice for mental illnesses [18, 19]. During the past years, mindfulness-based interventions (MBIs) have increasingly become a focus of interest due to their efficacy and low costs and low-threshold access for everyone. By combining elements of established cognitive behavioral therapy and psychoeducational content, MBIs aim to reduce stress-related symptoms and support a self-effective approach to improve mental and physical well-being [20]. MBIs are not only suitable for stress management, but can also be applied in clinical settings [15, 21]. In a comprehensive meta-analysis examining 12,145 patients with different psychological disorders, MBIs were at least as effective as cognitive behavioral therapy or pharmacological therapy [21]. Furthermore, MBIs have been shown to be most effective in patients with symptoms of depression and anxiety [22] and appear to be at least equivalent, if not superior, to classical psychotherapy in reducing depressive symptoms [23]. Various smaller studies demonstrate that MBIs also present a promising means of reducing anxiety and depression during the peripartum period [3, 24]. Apart from that, practicing MBIs may also positively influence physiological changes such as decrease of circulatory inflammatory proteins and reduction of cortisol response to stressors [25].

Considering the increasing omnipresence of the internet, MBI can also be offered electronically on a low-threshold and cost-effective basis via a (tablet) computer or smartphone (electronically based MBI = eMBI). Indeed, online-based psychological interventions were found to reduce symptoms of anxiety and depression as well as suicidal thoughts [26–28].

## Methods

### Study design

The *mindmom* study is a bicentric prospective randomized controlled trial (RCT) with a repeated measures and crossed-lagged panel design. Participants will be equally allocated to one of the two study arms: “8-week eMBI course” (intervention group) or “Treatment as usual (TAU) alone” (control group). Both groups will be compared regarding superiority of an electronic mindfulness-based intervention over TAU. The study is being conducted at the University women’s hospitals of Heidelberg and Tübingen, Germany. Ethics approval has been obtained from both universities.

#### Objectives

This study aims to examine the clinical effectiveness and cost-effectiveness of an eMBI (*mindmom* app) within a sample of pregnant women during the third trimester of pregnancy who were screened positive for emotional distress.

The following content-related hypotheses will be examined:

Participation in the intervention leads to (a) decrease of symptoms of depression/anxiety or lowers prevalence of peripartum depression, (b) decrease of neuroendocrine stress markers, (c) increase of quality of life, (d) promotion of a more positive perception of the birth experience and attachment to the newborn, and (e) a reduction in the number of cesarean sections.

### Ethics

The study was approved by the ethics committee of the Medical Faculty of the University of Heidelberg on 7 January 2019 (S-744/2018), and recruitment of study participants started in February 2019. Any protocol modifications will be communicated immediately. It is conducted according to the international standards of the Declaration of Helsinki and subsequent amendments. The study is performed in compliance with the study protocol and with good clinical practice guidelines with the aim of protecting and preserving human rights. Gathering and processing of all personal data are subject to confidentiality and the European General Data Protection Regulation (EU-GDPR). This study will follow the CONSORT statement (Consolidated Standards of Reporting Trials, http://www.consort-statement.org) and the SPIRIT guidelines (Standard Protocol Items: Recommendations for Intervention Trials) (Additional file 1) [29]. The study was registered in the German Clinical Trials Registry (DRKS 00017210, study protocol version 3.0 of 18 December 2018).

### Study population, recruitment, and eligibility criteria

All pregnant women, attending routine pregnancy care in the state of Baden-Wuerttemberg from the 13th up to the 28th week of gestation, are invited to participate in a screening for mental distress according to the Edinburgh Postnatal Depression Scale (EPDS) by their local gynecologists. The screening is administered while attending prenatal care either at a registered gynecologist or one of the study sites. All questionnaires are digitally transmitted and evaluated at one of the study centers (University of Heidelberg, Germany, or University of Tübingen, Germany). Women with an EPDS score > 9 are offered an interdisciplinary medical-psychological assessment, since this EPDS cutoff indicates the presence of emotional distress.

The assessment is conducted by a psychologist from the study team, either in person or by video consultation by using the screening items of the Structured Clinical Interview for DSM-5 (SCID-5-CV) [30].

Women with an increased level of emotional distress (EPDS score > 9) are eligible for study participation if they are over 18 years of age, are under 29 weeks of gestation, have sufficient German language skills, and have internet access. As the state of Baden-Wuerttemberg, Germany, is the model region for this new type of care, potential study participants must be insured with one of the participating health insurance providers. After the psychological evaluation, one of the study physicians obtained informed consent for trial participation.

Women are not eligible for study participation if they are expecting multiples and have an otherwise increased risk of preterm delivery (e.g., preterm rupture of membranes) or if a serious fetal malformation has been diagnosed that might require a medical intervention immediately after birth. In addition, acute psychotic episodes or diagnosed schizophrenic disorders, suicidality, substance abuse disorders, borderline personality disorder, bipolar disorders, traumatic experiences without reference to the current pregnancy, or the need for an acute psychiatric treatment constitutes exclusion criteria. Study participants should not have participated in any MBI during the current pregnancy.

### Randomization

After informed consent is obtained, the participants are randomized 1:1 into the intervention (IG = eMBI) or control group (CG = TAU). The randomization list was generated by an independent scientific assistant using the R package “blockrand,” v. 1.3 (R v. 3.4.2, Rstudio v. 1.1.383). The group variable is blinded with a binary code (“0” and “1”) that was determined prior to the first assessment and is only known to the study staff and the app developers. The randomization list was recorded in writing and uploaded to the electronic case report form (eCRF) [31].

### Sample size

Estimation of sample size and power analysis were calculated with G*Power (Version 3.1.9.2) [32, 33]. The estimation was implemented for the confirmatory hypotheses, which aim for interaction between the between- (group) and within-subject factor (time of measurement), i.e., that the symptom burden develops differently in the two study groups over the course of time. The analysis with the lowest power is the two-factorial ANOVA with the outcome “PRAQ” (5 measurements, see Table 3, hypotheses 4–6).

The estimation is based on type 1 error of α = .016, Bonferroni-adjusted for 3 confirmatory analyses, and a power of 1 − β = .90. The statistically significant effect size is based on the results of a pilot study on mindfulness-based prevention in pregnant women with a history of depression [34]. Based on the mean values, standard deviations, and case numbers reported in this study, a final treatment effect of Cohen’s *d* = .34 (intervention group: *M* = 4.90, *SD* = 5.22, *n* = 21; control group: *M* = 6.62, *SD* = 4.94, *n* = 29) is calculated for the outcome “depressive symptoms” (EDPS score). According to Cohen (1988), this can be classified as a small effect size. Thus, the number of cases for the ANOVA design is estimated for small treatment effects (*f* = .10). Thus, *N* = 196 cases are needed to evaluate the eMBI. Assuming a dropout rate of 30%, the number of cases was determined to be *N* = 280 to reach a case number of *N* = 196 (*n* = 140 for each group or per region and *n* = 70 per group and region). With a sample size of *N* = 196, large- (*f* = .40) and medium-sized (*f* = .25) effects can be detected with a power of 1 − β = .99 and .98, respectively. Only small effects (*f* = .10) cannot be ruled out sufficiently with a power of 1 − β = .27 in case of non-significant between-subject effects.

## Intervention

### Electronic mindfulness-based intervention

The eMBI presented here (*mindmom intervention*) was adapted to pregnancy by an interdisciplinary team of gynecologists, psychologists, and midwives and takes place between the 29th and 36th gestational weeks [35]. The eMBI comprises an established concept of eight weekly 45-min sessions [34] including psychoeducational content, approaches derived from cognitive behavioral therapy and mindfulness exercises especially adapted to pregnancy [36]. *Mindmom* teaches how to deal with stress, pregnancy- and birth-related anxiety, and perinatal depressive symptoms and supports the autonomy of the expectant mother with regard to the upcoming birth and the initial time with the baby. The app contains instructional videos, audio files, interactive worksheets, and a personal skills box in which the participant can collect exercises, texts, and videos that were perceived as helpful. Table 1 gives an overview of the contents of the *mindmom* sessions.

**Table 1:** Overview of the *mindmom* program

Module 1	Module 2	Module 3	Module 4	Module 5	Module 6	Module 7	Module 8
**Introduction** (the idea behind skill and mindfulness training)	**Fears and worries about birth/parenting**	**Coping with stress**	**Birth-related pain control**	**Birth**	**Me and my baby**	**Outlook puerperium**	**Graduation module—a review of all modules**

In order to improve adherence to the intervention, participants will be reminded by email about pending appointments and visits. Additionally, elements of gamification were implemented to support user compliance.

### Course of study

**Fig. 1:**
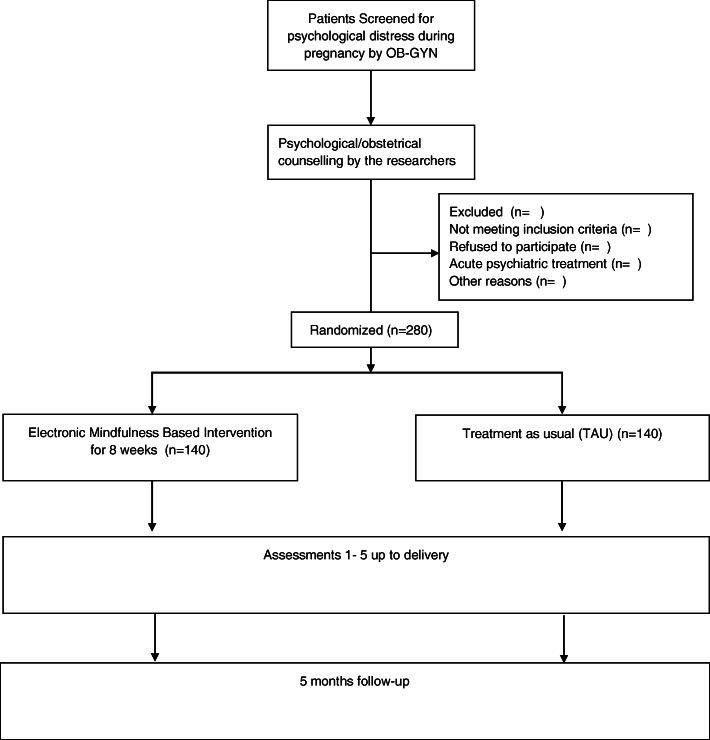
Flow chart of study

**Fig. 2:**
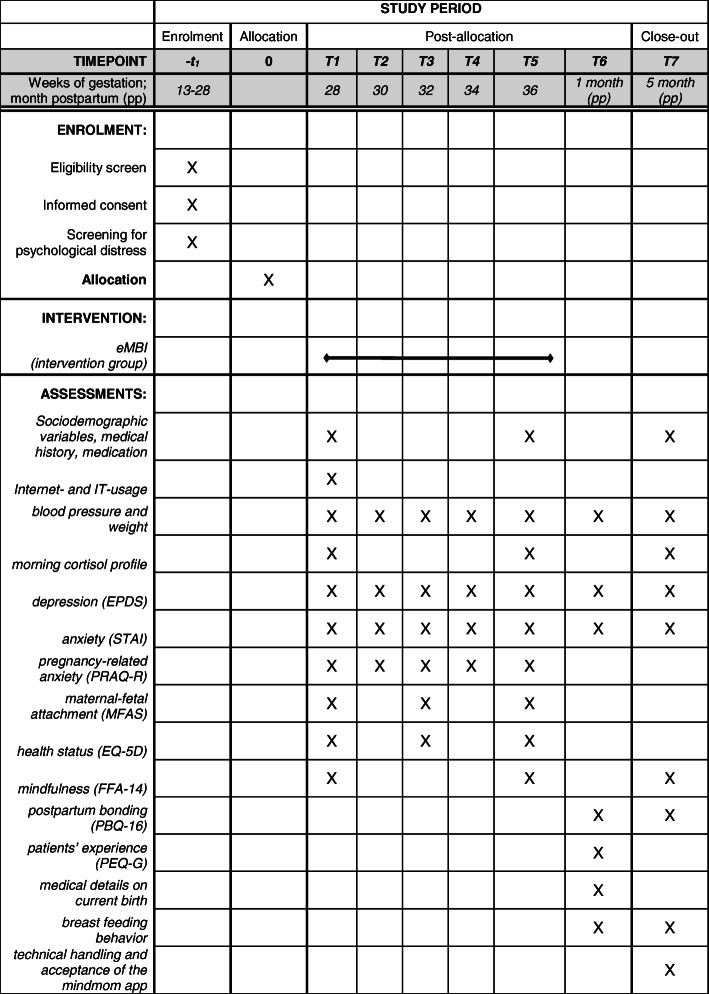
Schedule of enrolment, interventions, and assessments of the mindmom study

The beginning of the study is scheduled for the 29th gestational week. Every study participant receives access to the *mindmom* app. Participants of both groups (IG and TAU) are requested to complete digital assessments, which are scheduled every 2 weeks for the period of the intervention (visits 1–5), as well as 1 and 5 months postpartum (visits 6 and 7). The assessments are completed via the *mindmom* app and contain self-report questionnaires, sociodemographic and medical data, physiological measures, and morning cortisol profiles, which will be sent to the responsible study center by post. Table 2 provides an overview of the visit schedule and its content. Moreover, all study participants have access to an online pregnancy guidebook embedded in the mindmom app that provides valid information about pregnancy and birth on a weekly basis. Women in the TAU group are encouraged to participate in regular antenatal care and are conveyed to mental health care services, if required. The IG participates in the eMBI. Figure 1 shows the flow chart of the mindmom study. Figure 2 shows the schedule of enrolment, interventions, and assessments of the mindmom study.

**Table 2:** Study outcomes

	Time of assessment	Study outcomes	Instruments
**T1**	28th gestational week	• Symptoms of depression and (pregnancy-related) anxiety • Maternal-fetal attachment • Quality of life • Mindfulness • Morning cortisol profile • Sociodemographic variables (age, educational level, family status, income, occupation, number of children) • Medical history (gravidity, parity, fertility treatment) • Blood pressure, pulse, height, weight	EPDS, STAI, PRAQ-R MFAS EQ-5D FFA-14
**T2**	30th gestational week	• Symptoms of depression and (pregnancy-related) anxiety • Blood pressure, weight	EPDS STAI, PRAQ-R
**T3**	32nd gestational week	• Symptoms of depression and (pregnancy-related) anxiety • Maternal-fetal attachment • Quality of life • Blood pressure, weight	EPDS STAI, PRAQ-R MFAS EQ-5D
**T4**	34th gestational week	• Symptoms of depression and (pregnancy-related) anxiety • Blood pressure, weight	EPDS STAI, PRAQ-R
**T5**	36th gestational week	• Symptoms of depression and (pregnancy-related) anxiety • Maternal-fetal attachment • Quality of life • Mindfulness • Morning cortisol profile • Blood pressure, weight	EPDS STAI, PRAQ-R MFAS EQ-5D FFA
**T6**	1st month postpartum	• Symptoms of depression and anxiety • Postpartum bonding • Satisfaction with medical care around the birth • Satisfaction birth • Breast feeding behavior • Blood pressure, weight • Medical details on current birth	EPDS, STAI PBQ-16 PEQ-G
**T7**	5th month postpartum	• Symptoms of depression and anxiety • Postpartum bonding • Mindfulness • Blood pressure, weight • Breastfeeding behavior • Morning cortisol profile • Technical handling and acceptance of the *mindmom* app	EPDS, STAI PBQ-16 FFA-14
**T1–7**	Continuously	• Out-of-pocket payments for alternative diagnostic and therapeutic methods, homeopathic products or non-prescription drugs	Cost diary

### Primary outcomes

#### The Edinburgh Postnatal Depression Scale

Symptoms of depression during pregnancy are assessed with the German version of the EPDS [37, 38]. The EPDS is a 10-item questionnaire that is used as a screening instrument and for research purposes. Item scores are summed up to a total score, with higher scores indicating a higher symptom burden. At a cutoff value of 9 (EPDS > 9), the sensitivity of detecting a clinically significant depression is 0.96, the specificity is 1.00, and the positive predictive value is 1.00 [37–39]. The scale has a good internal consistency (Cronbach’s α = 0.81).

#### State-Trait Anxiety Questionnaire

Symptoms of anxiety are assessed according to the German version of the State-Trait Anxiety Questionnaire (STAI), a 40-item questionnaire based on Cattell’s model of anxiety (Laux et al. [40]; Spielberger et al. [41]). The STAI consists of 2 subscales separately assessing anxiety as both a general trait and a temporary condition, which may be used together or separately, each showing an excellent internal consistency (Cronbach’s α = .90) [40–42].

#### Pregnancy-Related Anxiety Questionnaire

Pregnancy-related anxiety is measured with the German abridged version of the Pregnancy-Related Anxiety Questionnaire (PRAQ-R) [43–45]. The PRAQ-R consists of 10 items with three subscales (birth anxiety, fear of having a physically or mentally ill child, and worrying about one’s own physical appearance). The internal consistency for global scale is good [44].

### Secondary outcomes

#### Perceived attachment towards the fetus

The attachment to the unborn child is evaluated by using the revised German version of the Maternal-Fetal Attachment Scale (MFAS) [44, 46]. The MFAS is a 24-item questionnaire with 4 subscales (anticipate interaction with the baby, personal involvement, name the baby, and interaction with the fetus). The internal consistency of the questionnaire is acceptable with a Cronbach’s alpha of 0.792.

#### Postpartum Bonding Questionnaire

The quality of postpartum bonding is evaluated by applying the abridged version of the Postpartum Bonding Questionnaire (PBQ-16) [47, 48]. The PBQ-16 consists of 16 items and has a good internal consistency (Cronbach’s α = .85).

#### Quality of life

Health-related quality of life is measured with the EQ-5D-5L, a generic instrument that was developed by the EuroQol Group [49–51]. The internal consistency is good (Cronbach’s alpha of 0.83). Several studies show that EQ-5D is a valid instrument during the peripartum period, too [51].

#### Mindfulness

Individual mindfulness as a personal trait is evaluated by using the abridged version of the “Freiburger Fragebogen zur Achtsamkeit” (FFA-14 questionnaire) [52, 53]. The internal consistency is good (Cronbach’s α = .86).

#### Birth experience

Satisfaction with birth-related medical care (medical care, midwives, nursing staff, and service) is assessed with the Patients’ Experience Questionnaire Birth (PEQ-G). The PEQ-G consists of 18 items and has an excellent internal consistency (Cronbach’s α = 0.9102) [54].

#### Cortisol profile

Cortisol awakening response is measured by saliva samples (*Awakening Response*) in order to assess individual stress levels objectively [55].

#### Measures for cost-effectiveness

Health care service use and costs for both groups will be measured by means of secondary claims data of the statutory health insurers. This includes direct costs such as general and specialist care, medication, hospitalization, therapeutic products, medical aids, and other services related to pregnancy as well as costs associated with absenteeism. Further, the out-of-pocket costs by families will be assessed using a specific self-completing cost diary.

### Risk-benefit assessment

We do not expect any major risks associated with study participation for the pregnant woman or the fetus. The radiation exposure due to the use of the app is negligible. A detailed risk assessment is carried out as part of the psychological assessment. If acute suicidal tendency is detected by the EPDS screening during study participation, the responsible coordinating body will be alerted. If the study participant feels emotionally burdened, for example, by attending the regular visits, contact with the coordinating office can be made at any time. The interests of study participants always have priority. If participation in the study causes any kind of psychological stress, participation can be terminated at any time.

### Data collection and management

Data will be collected via (1) the mindmom app based on electronic Patient-Reported Outcomes (ePRO) and (2) entries of the study staff to the electronic case report (baseline anamnesis and physiological data). Participants will receive computer-generated emails or smartphone reminders to complete questionnaires. These reminders will occur at baseline and 2, 4, 6, and 8 weeks. Data storage and transfer are encrypted. Plausibility check is carried out as soon as the data are entered into the system. Data entry is protected by individual access data for each member of the study staff. All participants receive pseudonymized study IDs by the coordinating bodies. The study key is encrypted, is only accessible by the study staff and the developers of the application, and will be deleted after study completion. Information gathered with the mindmom app is regularly and incrementally merged with the information provided by the eCRF at the evaluating institute via the pseudonymized study IDs. For research data analysis, only pseudonymized data will be used meaning that identifying data is separated from medical data. If data is shared, it will only be shared without the participants’ identifier. A transfer of scientific data to third parties (e.g., the “Open Science Framework Repository”; OSF; https://osf.io/) will only contain anonymized data. The collected primary data will be linked to the secondary claims data provided by the statutory health insurers. To ensure data security, patient data will be pseudonymized by means of the predefined study IDs and appropriate systems for data transfer will be used.

### Data analyses

The results will be reported following CONSORT recommendations [29]. All statistical analyses are conducted by using IBM® SPSS® and AMOS® (current version 26.0.0). Sociodemographic and clinical data will be described by means of frequencies and percentages or means and standard deviations. Confirmatory and exploratory research hypotheses will be analyzed using multivariate analysis of variance with repeated measurements, correlation analysis, and chi-square tests (see Table 3).

**Table 3:** Analytic procedure

	Statistical test	Number of tests	Test statistic	Relevant effect
**Confirmative hypotheses**				
**1–3**	2 (group) × 7 (measurement)—ANOVA for repeated measures on the factor “measurement”	2	*F*-distribution	Group × measurement interaction effect
**4–6**	2 (group) × 5 (measurement)—ANOVA for repeated measures on the factor “measurement”	1	*F*-distribution	Group × measurement interaction effect
**Exploratory hypotheses**				
**1–9 and 14–16**	2 (group) × 3 (measurement)—ANOVA for repeated measures on the factor “measurement”	4	*F*-distribution	Group × measurement interaction effect
**10–12**	2 (group) × 2 (measurement)—ANOVA for repeated measures on the factor “measurement”	1	*F*-distribution	Group × measurement interaction effect
**13**	*t* test for independent samples	1	*t*-distribution	Group main effect
**14**	One-dimensional *χ*^2^ test with 2 cells	3	*χ*^2^-distribution	Group main effect

The critical, local α-errors for the 3 tests evaluating the confirmatory hypotheses are Bonferroni-adjusted to α_local_ = .05/3 = .016 to avoid exceeding a global α-error of α_global_ = .05 by performing multiple tests. For the exploratory analyses, the critical, local α-errors are not adjusted and set to a conventional level of α_local_ = .05. Owing to inflation of the global α-error by multiple testing (9 tests) to α_global_ = 1 − (1 − 0.05)^9^ = 0.37, the exploratory analyses have a descriptive character and will be used to generate future hypotheses.

Dunn’s multiple comparison procedure is used as a post hoc test because it allows hypothesis-driven and economic multiple testing. This procedure results in a minimum significant difference (ψ). The test value is t-distributed. As effect sizes, partial η^2^ and ω^2^ are used. These are sample-based or population-based estimators of explained variances, respectively. According to Cohen [56], η^2^/ω^2^ = .01 represents small, η^2^/ω^2^ = .06 represents medium, and η^2^/ω^2^ = .14 represents large effects.

Furthermore, identified relationships are tested for causality. This can be done by means of the study’s cross-lagged panel design (outcomes and confounding variables are collected at multiple measurement times). Here, linear structural equation modeling is used.

In order to clarify the mediating mechanisms as well as the modifiability of the identified associations, additional explorative path analyses are performed on mediating and moderating processes according to Hayes’ regression-based approach [57] using the SPSS®—macro “PROCESS” (current v. 3.4).

Moreover, data will be analyzed by multi-level modeling. Hereby, the repeated measures (level 1) will be nested in the patients (level 2). A simulation study shows that only small samples on level 2 (< *n* = 50) lead to biased estimations of the standard errors on level 2 [58]. Expecting invariant results, the exploratory application of these models can increase the assurance regarding the exclusion of small between subject effects.

In accordance with the ICH Guideline E9, the primary analyses are performed according to the intention-to-treat (ITT) principle. Per-protocol (PP) analyses on sensitivity of effects are implemented in an exploratory fashion.

In order to reliably estimate missing values and thus to protect the analyses against response and selection biases, multiple imputations will be used [59]. First of all, missing values will be tested by Little’s Missing-Completely-At-Random test [60]. Following a negative result, *n* = 20 data sets with all variables of the analytic model are estimated. Hereby, value estimations are restricted to scale-specific ranges. Results are then pooled over the *n* = 20 data sets.

In accordance with the methods proposed by the German Institute for Quality and Efficiency in Health Care (IQWiG), the cost-effectiveness analysis will be carried out from the perspective of the statutory health insurance and, if possible, additionally include a societal and/or individual perspective [61]. The incremental cost-effectiveness ratio (ICER) will be calculated dividing the difference in costs by the difference in health benefit of the intervention compared to standard care. Costs are assessed in monetary units, and the benefit is measured by the primary (severity of symptoms) and secondary outcomes (f. e. cesarean sections, health related quality of life). The perspective of the analysis thereby determines the relevant cost components.

### Data monitoring

Data will be monitored regularly. Sociodemographic variables are descriptively monitored on statistical parallelism with respect to mean values and standard deviations (e.g., age) or frequencies (e.g., level of education attained) to ensure comparability between the two groups. Reports from administrative monitoring are given at regular intervals.

In addition to administrative monitoring (including checks for recruitment rates), confirmatory monitoring will be carried out to ensure the study’s feasibility. *K* = 3 interim analyses (interval: *n* = 94 recruited cases) will be performed with an α-error adjusted according to Lan and Demets [62].

The independence of the evaluators is ensured via institutional and spatial segregation as well as by pseudonymization of participant data and blinding of study groups. Influences by consortia members or the sponsors on methodological procedures, data processing, and presentation of results are foreclosed. The evaluators will publish interim and final results at relevant scientific conferences and in relevant scientific peer-reviewed journals.

## Discussion

Mental disorders, especially anxiety and depressive disorders, are common during the peripartum period and may have far-reaching consequences for both mother and child. MBIs offer a promising approach for reducing symptoms of depression and anxiety and preventing postnatal depression. Due to the high number of screened patients and associated diagnoses according to DSM/ICD criteria, the study will provide valuable data on the prevalence of peripartum stress and mental disorders. If the hypothesized results are obtained, this intervention may offer an important option for psychologically distressed women during pregnancy. Electronic-based solutions may reduce geographic, financial, and psychological limitations. The *mindmom* app thus represents a promising approach to provide effective, low-threshold, and cost-effective help for pregnant women with psychological distress and may thereby reduce the negative impact on perinatal health outcome. As a unique strength of the study, this trial combines an analysis of self-assessments, physiological data such as saliva cortisol and blood pressure, and insurance claims data. To our knowledge, no other trial is currently being conducted by combining these elements. Acknowledged study limitations include the possibility that participants in the intervention and the TAU group could seek out other psychological and/or pharmacological interventions, which cannot be discouraged for ethical reasons.

## Supplementary Information

**Additional file 1.** SPIRIT 2013 Checklist: Recommended items to address in a clinical trial protocol and related documents

## Data Availability

Not applicable.
