# Supplementary material for: Effectiveness and cost-effectiveness of an electronic mindfulness-based intervention (eMBI) on maternal mental health during pregnancy: the mindmom study protocol for a randomized controlled clinical trial
Source: Trials. 2020 Nov 17;21:933. doi: 10.1186/s13063-020-04873-3 (PMC7672841; doi:10.1186/s13063-020-04873-3)
Supplement: Supplementary file 1 — Additional file 1. SPIRIT 2013 Checklist: Recommended items to address in a clinical trial protocol and related documents [file 13063_2020_4873_MOESM1_ESM.doc]

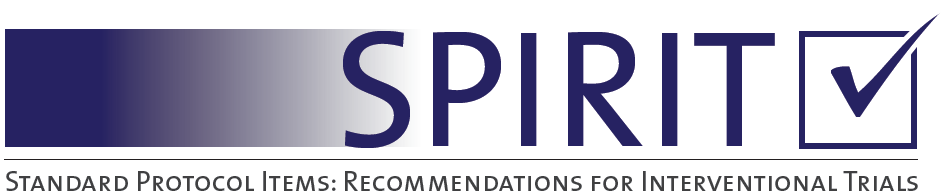


SPIRIT 2013 Checklist: Recommended items to address in a clinical trial protocol and related documents*

| Section/item | ItemNo | page, more information |
| --- | --- | --- |
| **Administrative information** | | |
| Title | 1 | title identifying the study design, intervention and trial acronym  page 1 |
| Trial registration | 2a  2b | trial identifier and registry name  Trial registration: DRKS00017210  registry name: Deutsches Register Klinischer Studien  <https://www.drks.de/drks_web/navigate.do?navigationId=trial.HTML&TRIAL_ID=DRKS00017210>  page 3 |
| Protocol version | 3 | date and version identifier  page 6 |
| Funding | 4 | source of financial funding  page 15 |
| Roles and responsibilities | 5a | Names and affiliations of protocol contributors: page 1  roles of protocol contributors: page 15 |
|  | 5b | Name of the trial sponsor  page 15 |
|  | 5c | Role of study sponsor/funder in study design, data collection, analysis, and interpretation of data; writing of the report.  page 15 |
|  | 5d | Composition, roles, and responsibilities of the coordinating centre, steering committee, endpoint adjudication committee, data management team, and other individuals or groups overseeing the trial, if applicable (see Item 21a for data monitoring committee)  page 15 |
| Introduction |  |  |
| Background and rationale | 6a | Description of research question and justification for undertaking the trial, including summary of relevant studies  page 4-5 |
|  | 6b | Explanation for choice of comparators  page 5-6 |
| Objectives | 7 | Specific objectives or hypotheses  page 6 |
| Trial design | 8 | Description of trial design including type of trial (RCT), and framework (superiority)  page 5-6 |
| Methods: Participants, interventions, and outcomes | | |
| Study setting | 9 | Description of study setting, study sites  page 6-7 |
| Eligibility criteria | 10 | Inclusion and exclusion criteria for participants, individuals who will perform the interventions  page 6-7 |
| Interventions | 11a | Interventions for each group with sufficient detail to allow replication, including how and when they will be administered  page 8-9  Figure 1 |
|  | 11b | Criteria for discontinuing intervention for a given trial participant  page 11 |
|  | 11c | Strategies to improve adherence to intervention protocol  page 8 |
|  | 11d | Relevant concomitant care and interventions that are permitted or prohibited during the trial  non applicable, no concomitant care or interventions prohibited during the trial. |
| Outcomes | 12 | Primary, secondary, and other outcomes, including the specific measurement variables analysis metric  page 9-11  table 2 |
| Participant timeline | 13 | Time schedule of enrolment, interventions, assessments, and visits for participants.  page 9  figure 1 |
| Sample size | 14 | Estimated number of participants needed to achieve study objectives and how it was determined, including clinical and statistical assumptions supporting any sample size calculations  page 7-8 |
| Recruitment | 15 | Strategies for achieving adequate participant enrolment to reach target sample size  page 6-7 |
| **Methods: Assignment of interventions (for controlled trials)** | | |
| Allocation: |  |  |
| Sequence generation | 16a | Method of generating the allocation sequence  page 7 |
| Allocation concealment mechanism | 16b | Mechanism of implementing the allocation sequence (eg, central telephone; sequentially numbered, opaque, sealed envelopes), describing any steps to conceal the sequence until interventions are assigned  Not applicable |
| Implementation | 16c | Who will generate the allocation sequence, who will enrol participants, and who will assign participants to interventions  page 7 |
| Blinding (masking) | 17a | page 7 |
|  | 17b | If blinded, circumstances under which unblinding is permissible, and procedure for revealing a participant’s allocated intervention during the trial  not applicable |
| **Methods: Data collection, management, and analysis** | | |
| Data collection methods | 18a | Plans for assessment and collection of outcome, baseline, and other trial data; page 11-12.  description of study instruments along with their reliability and validity;  page 9-11 |
|  | 18b | Plans to promote participant retention and complete follow-up, including list of any outcome data to be collected for participants who discontinue or deviate from intervention protocols  page 12 |
| Data management | 19 | Plans for data entry, coding, security, and storage, including any related processes to promote data quality.  page 11-12 |
| Statistical methods | 20a | Statistical methods for analysing primary and secondary outcomes.  page 12-13  table 3 |
|  | 20b | Methods for any additional analyses  page 12-13  table 3 |
|  | 20c | Definition of analysis population relating to protocol non-adherence, statistical methods to handle missing data  page 13 |
| **Methods: Monitoring** | | |
| Data monitoring | 21a | page 13-14 |
|  | 21b | Description of any interim analyses and stopping guidelines  page 13-14 |
| Harms | 22 | Plans for assessing, reporting, and managing solicited and spontaneously reported adverse events of trial intervention  page 11 |
| Auditing | 23 | Frequency and procedures for auditing trial conduct, if any, and whether the process will be independent from investigators and the sponsor  page 14 |
| Ethics and dissemination | | |
| Research ethics approval | 24 | Ethics approval  page 14 |
| Protocol amendments | 25 | Plans for communicating important protocol modifications (eg, changes to eligibility criteria, outcomes, analyses) to relevant parties (eg, investigators, REC/IRBs, trial participants, trial registries, journals, regulators)  page 6 |
| Consent or assent | 26a | Who will obtain informed consent or assent from potential trial participants or authorised surrogates, and how (see Item 32)  page 7 |
|  | 26b | Additional consent provisions for collection and use of participant data and biological specimens in ancillary studies, if applicable  Not applicable, no ancillary studies planned |
| Confidentiality | 27 | How personal information about potential and enrolled participants will be collected, shared, and maintained in order to protect confidentiality before, during, and after the trial  page 6 |
| Declaration of interests | 28 | Financial and other competing interests for principal investigators for the overall trial and each study site  page 14 |
| Access to data | 29 | Statement of who will have access to the final trial dataset, and disclosure of contractual agreements that limit such access for investigators  Any data required to support the protocol can be supplied on request.  Page 11-12 |
| Ancillary and post-trial care | 30 | Provisions, if any, for ancillary and post-trial care, and for compensation to those who suffer harm from trial participation  Not applicable, no provisions planned, no major risks associated with study participation |
| Dissemination policy | 31a | Plans for investigators and sponsor to communicate trial results to the public, and other relevant groups (eg, via publication, reporting in results databases, or other data sharing arrangements)  Page 11-12 (data collection, management and analyses)  Page 13-14 (data monitoring) |
|  | 31b | Authorship eligibility guidelines and any intended use of professional writers  All named authors adhere to the authorship guidelines of Trials. All authors have agreed to publication.  Page 15 |
|  | 31c | Plans, if any, for granting public access to the full protocol, participant-level dataset, and statistical code  The datasets analysed during the current study are available from the corresponding author on reasonable request. |
| Appendices |  |  |
| Informed consent materials | 32 | Model consent form and other related documentation given to participants and authorised surrogates  The consent form and materials are available from the corresponding author on request. |
| Biological specimens | 33 | Plans for collection, laboratory evaluation, and storage of biological specimens for genetic or molecular analysis in the current trial and for future use in ancillary studies, if applicable  not applicable, no collection or storage of biological specimens for genetic/molecular analysis planned. |
